# Nasal Absorption of Macromolecules from Powder Formulations and Effects of Sodium Carboxymethyl Cellulose on Their Absorption

**DOI:** 10.1371/journal.pone.0159150

**Published:** 2016-09-06

**Authors:** Akiko Tanaka, Tomoyuki Furubayashi, Akifumi Matsushita, Daisuke Inoue, Shunsuke Kimura, Hidemasa Katsumi, Toshiyasu Sakane, Akira Yamamoto

**Affiliations:** 1 Department of Biopharmaceutics, Kyoto Pharmaceutical University, Misasagi, Yamashina, Kyoto 607–8414, Japan; 2 School of Pharmacy, Shujitsu University, Nishikawara, Kita, Okayama 703–8516, Japan; 3 Faculty of Pharmaceutical Sciences, Doshisha Women’s College of Liberal Arts, Kodo, Kyotanabe, Kyoto 610–0395, Japan; 4 Department of Pharmaceutical Technology, Kobe Pharmaceutical University, Motoyamakita-machi 4-19-1, Higashinada-ku, Kobe 658–8558, Japan; Laurentian, CANADA

## Abstract

The nasal absorption of macromolecules from powder formulations and the effect of sodium carboxymethyl cellulose (CMC-Na) as a pharmaceutical excipient on their absorption were studied. Model macromolecules were fluorescein isothiocyanate-labeled dextran (average molecular weight of 4.4kDa, FD4) and insulin. The plasma concentration of FD4 after application of the powder containing 50% starch (control) was higher than that after application of the solution, and the absorption from 50% starch powder was enhanced by the substitution of starch with CMC-Na. The fractional absorption of FD4 after administration of the CMC-Na powder formulation was 30% and 40% higher than that after administration from the solution and the starch powder, respectively. The nasal absorption of insulin from the powder and the effect of CMC-Na were similar with those of FD4. The effective absorption of FD4 and insulin after application of powder with CMC-Na could be due to the increase in the nasal residence of FD4 and insulin. No damage in the nasal mucosa or dysfunction of the mucociliary clearance was observed after application of the drug powder and CMC-Na. The present findings indicate that nasal delivery of powder formulations with the addition of CMC-Na as an excipient is a promising approach for improving the nasal absorption of macromolecules.

## 1. Introduction

Peptide and protein drugs are a currently popular and effective treatment for various diseases. Because of the poor absorption of peptides and proteins from the gastrointestinal tract, a subcutaneous injection has been the preferred route of administration of such drugs. However, this route is associated with poor patient compliance and QOL because of the pain caused by injection and the risk of inflammation and infection. Therefore, a new delivery system of peptide and protein drugs is highly desirable for the improvement of compliance and QOL of patients.

It was reported that peptide and protein drugs are well absorbed from the nasal cavity as compared to the oral route, because of the highly developed vasculature with wide fenestrae under the nasal epithelia [1]. Additionally, the first-pass effect associated with hepatic metabolism can be avoided through the nasal route [2]. Among the various strategies available, the nasal cavity has now been recognized as a very promising administration route for the systemic drug delivery of peptides and proteins. Therefore, many researchers have focused on and reported the absorption of peptide and protein drugs after nasal administration [3–5]. However, the nasal absorption of peptides and proteins is still poor compared to absorption through subcutaneous injection, because of the rapid mucociliary clearance limiting the nasal residence of the drug [6–8], the enzymatic degradation, and the small surface area of the nasal epithelium.

In most research on nasal drug absorption so far, liquid formulations such as solution, emulsion, and suspension have been used [9–12]. As compared to liquid formulations, there are many advantages of the powder formulation, such as the better stability of the solid drug, application of larger dose, and the higher concentration of the drug in the nasal mucosa [13–16]. In spite of such merits of powder formulations, few reports have described the nasal drug absorption of macromolecules from powder. Therefore, the first purpose of this study was to examine the absorption of macromolecules after nasal application of their powder formulation.

Pharmaceutical excipients are usually added to most powder formulations. For example, lactose is often used as a diluent. Cellulose derivatives such as carboxymethyl cellulose (CMC-Na), hydroxylpropyl cellulose (HPC), and hydroxypropylmethyl cellulose (HPMC) are usually used as a binder. These excipients are added for granulation and tablet manufacturing. The effect of the excipient on the nasal drug absorption is likely marked in comparison with absorption after oral administration, since the powder formulation is directly applied onto the nasal mucosa. The second purpose of this study was to clarify the effect of excipients on the nasal absorption of macromolecules from the powder to which the excipient is added. This study focused on CMC-Na, a typical binder [17, 18]. Since the dissolution of CMC-Na in the nasal cavity increases the viscosity of the formulation, it may expectedly improve the nasal drug absorption.

In this study, the absorption of the model macromolecules isothiocyanate-labeled dextran (average molecular weight of 4.4 kDa, FD4) and insulin was examined after nasal application of the powder to rats. At the same time, the absorption of macromolecules from the powder to which CMC-Na is added was evaluated and compared that from the liquid formulation and the control powder formulation.

## 2. Materials and Methods

### 2.1 Materials

Glucose CII-test Wako, an insulin enzyme immunoassay kit, LDH-cytotoxic Wako, and mucin from pig stomach were purchased from Wako Pure Chemical Industries, Ltd. (Osaka, Japan). Porcine Insulin (specific activity, 27 U/mg), fluorescein isothiocyanate-labeled dextran (FD4, average MW: 4.4 kDa), and fluorescent microspheres (FMS; Fluoresbrite^®^ YG microspheres, size 6 μm) were supplied by NACALAI TESQUE, Inc. (Kyoto, Japan), Sigma-Aldrich Co. (St. Louis, MO, USA), and Polysciences Inc. (Warrington, PA, U.S.A.), respectively.

### 2.2 *In vivo* nasal absorption

#### 2.2.1 Animals and ethical approval

Male Wister rats (Shimizu Laboratory Supplies, Kyoto, Japan) weighing 220–250 g were used in all animal experiments. They were housed in group cages (n = 3) in a room with controlled temperature, a 12:12 h light-dark cycle, and free access to water and food. All animal studies were previously approved by the Animal Ethics Committee at Kyoto Pharmaceutical University (Permit Number: 15-12-074), and carried out in accordance with the guidelines. All surgery was performed under pentobarbital sodium anesthesia, and all efforts were made to minimize suffering of the animal.

#### 2.2.2 Preparation of liquid and powder formulations

Liquid formulations: FD4 was dissolved in phosphate-buffered saline (PBS) at a concentration of 125 mg/mL. Because the solubility of insulin is not high enough to dissolve at a concentration of 500 μg/5 μL, insulin was suspended in PBS at 100 mg/mL.

Powder formulations: The drug powder was mixed with starch or CMC-Na at weight ratio of 1:1.

#### 2.2.3 Animal study

Rats were anesthetized with intraperitoneal pentobarbital sodium (52 mg/kg), and the right femoral artery was cannulated with polyethylene tubing. Dry powder formulation (Insulin: 1 mg, FD4: 10 mg) was administered into the nasal cavity with a special device. The powder was placed into a polyethylene tube connected to a disposable micropipette tip. The powder was dispersed into the nasal cavity by releasing the air compressed in a syringe by opening a three-way stopcock connecting the disposable tip and the syringe. The dose of the drug was determined from the difference in the weight of the polyethylene tube and disposable tip before and after administration, because the powder easily adsorbed to the tip through the electrostatic force. The liquid formulation (5 μL) was administered into the nasal cavity with a micropipette. After the administration, the animal was kept conscious in a cage (KN-326-III, Natsume, Tokyo, Japan) throughout the experiment. Blood samples were collected at intervals for 360 min after drug administration. Blood samples were centrifuged at 13,000 rpm for 5 min to obtain the plasma. Plasma samples were stored frozen at -40°C until assay.

### LDH activity

i Cannulation group (single administration)

The esophagus and trachea of the rat were surgically operated on according to the method of Hirai et al. [19]. The nasopalatine was closed with a surgical adhesive. Starch (1 mg), CMC-Na (1 mg), or PBS (5 μL) was dispersed or instilled into the nostril. The nasal lavage fluid was collected 6 h after administration.

ii No-cannulation group (single administration)

Starch (1 mg), CMC-Na (1 mg), or PBS (5 μL) was sprayed or instilled into the nostril. Using the method of Hirai et al., the esophagus and trachea were surgically operated on 6 h after administration, as described above.

iii Repeated administration

Starch (1 mg), CMC-Na (1 mg), or PBS (5 μL) was dispersed or instilled into the nostril once a day for 7 d. On the day following the last application, the esophagus and trachea were surgically operated on as described above.

After the pretreatment described above, the nasal cavity was washed with 8 mL PBS through the cannula placed in the esophagus. The activity of LDH in the nasal lavage fluid was determined using LDH-cytotoxic Wako.

### 2.5 Mucociliary Clearance

#### 2.5.1 Preparation of Mucin Solution

Mucin from the pig stomach was used in order to supplement the excised nasal septum with mucus. Mucin was suspended in Hank’s balanced salt solution (HBSS) at a concentration of 2 w/v%, and stirred well. The resulting suspension was centrifuged twice at 13,000 rpm for 20 min to remove insoluble debris. The obtained supernatant was stored at 4°C and used as mucin solution (MS).

#### 2.5.2 *In vitro* evaluation of the mucociliary clearance

Mucociliary clearance was measured according to the method reported previously [20]. The rat nasal septum was excised surgically from the rat under pentobarbital anesthesia. The movement of FMS on the surface of the nasal septum was observed with a fluorescent microscope 30 s after the instillation of the FMS suspension, and photographs were taken serially with a CCD camera (CV-20M, Hitachi, Tokyo, Japan) at 3 s intervals for up to 5 min. The moving velocity of FMS was calculated from the traveling distance of FMS determined from serial photographs.

### 2.6 Assay of the drug

For determination of FD4 concentrations in the plasma, acetonitrile (100 μL) was added to the plasma (100 μL) for deproteination. The mixture was vortexed and centrifuged at 13,000 *g* for 5 min. The supernatant (100 μL) was analyzed with a spectrofluorometer at excitation wavelength of 485 nm and emission wavelength of 535 nm.

The nasal absorption of insulin was evaluated by measuring the insulin and glucose levels in the plasma [21–23]. The concentration of insulin and glucose in the plasma was determined with insulin-EIA TEST and Glucose CII-test Wako, respectively.

### 2.7 Calculation of the fraction absorbed

The fractional absorption of FD4 was calculated based on the deconvolution from zero to the final sampling time using WinNonlin (Certara G.K., Tokyo, Japan).

### 2.8 Data calculation

The concentration of endogenous insulin before insulin application was subtracted from concentrations of insulin in the plasma to precisely evaluate the absorption of insulin.

Because of the powder sticking to the device tip by electrostatic charge, individual doses of FD4 and insulin after powder administration were low. For example, doses of insulin administered as a powder, which are listed in Table 1, are approximately 70% of the prepared amount (500 μg). Therefore, concentrations of FD4 and insulin in the plasma were normalized with the dose in each rat, and are indicated as %dose/mL and μU/mL/g insulin, respectively.

**Table 1:** Pharmacokinetic parameters of insulin and glucose in the plasma after nasal administration of suspension, starch powder, and CMC-Na powder.

Formulation	AUC_insulin, 0→180_	AAC_glucose, 0→180_	D (%)	Dose of insulin
	(μU・min/mL)	(%・min)		(μg)
Suspension	5758 ± 2387	1602.0 ± 477.4	8.9 ± 2.7	500
Starch powder	8420 ± 1505	1894.8 ± 779.8	15.0 ± 6.8	335 ± 20.2
CMC-Na powder	9331 ± 312	2380.5 ± 570.3	20.2 ± 5.4	373 ± 26.2

Results are expressed as the mean ± S.E. of at least three experiments.

The area above the curve of glucose (AAC_glucose_) is the index of the *in vivo* pharmacological potency of insulin [24] and is defined as the area between the curve of plasma glucose and the horizontal line of the initial glucose level (100%). AAC_glucose_ and the area under the curve of insulin (AUC_insulin_) were calculated by the trapezoidal rule from zero to the final sampling time (180 min). The decrement of plasma glucose level (*D*%) after intranasal administration was calculated based on the modified method as described previously [23, 25]. The difference in the dose in the individual rat was corrected using the following equation.

$$D\;(\%)=\frac{AAC_{\text{glucose}}{}_{,\;0\to 180}(\%\cdot \text{min})\times D\;(\mu g)/D_{ind}\;(\mu \text{g})}{180\;(\min)}$$

where *D* and *D*ind are the dose administered as suspension (500 μg) and the dose in the individual rat to which the powder was administered, respectively.

### 2.9 Statistical analysis

All the experiments in the study were performed at least in triplicate, and the data are expressed as the mean ± standard error (SE). The statistical comparisons were conducted using an analysis of variance with subsequent Dunnett’s multiple comparison tests.

## 3. Results

### 3.1 The comparison of the nasal absorption of FD-4 from the solution and powder formulations in rats

**Fig 1:**
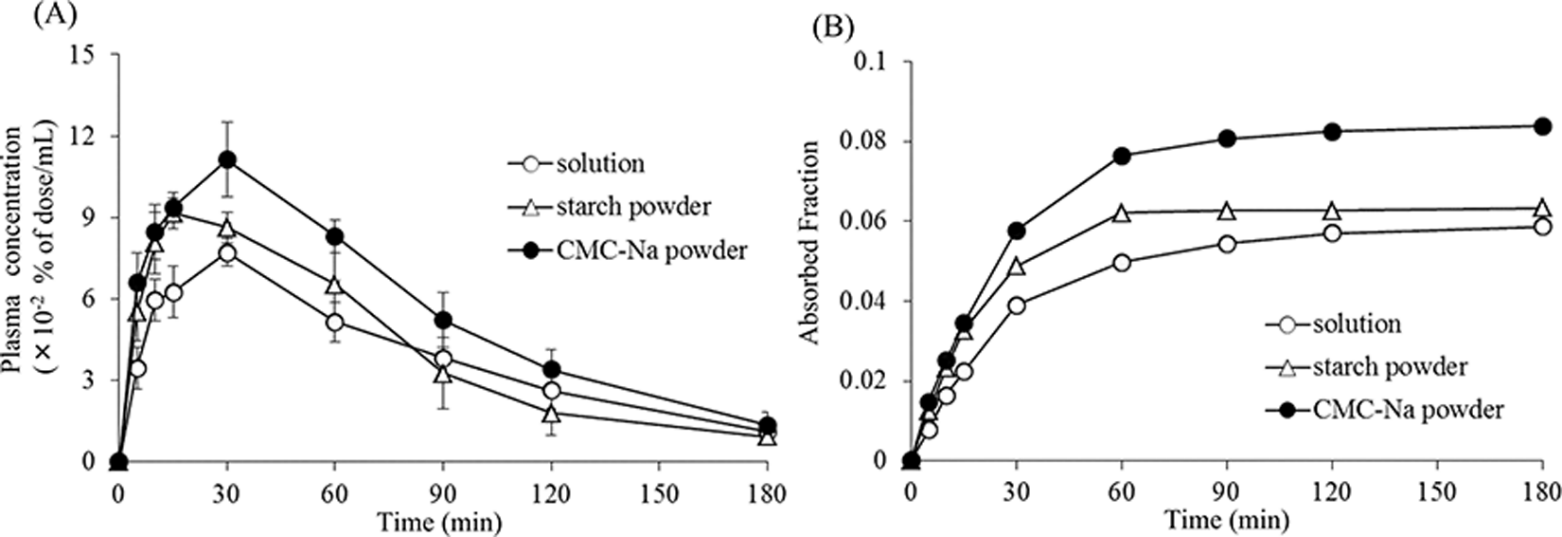
(A) The concentration of FD4 in the plasma and (B) changes in the fraction of FD4 absorbed after nasal administration of various formulations to rats. Keys; ○, solution; △, starch powder; ●, CMC-Na powder. Results are expressed as the mean ± S.E. of at least three experiments.

Fig 1A shows the plasma concentration-time profiles of FD4 after nasal administration of various formulations in rats. The powder formulation containing starch served as a control of powder formulations, because starch does not dissolve in water and showed no effect on the nasal mucosa. As shown in Fig 1A, a higher plasma concentration of FD-4 from both powder formulations was detected, as compared with that after administration of the solution. Additionally, the nasal absorption of FD-4 from the CMC-Na formulation was higher than that from the starch formulation. The change of the fractional absorption of FD-4 is shown in Fig 1B. In comparison with the fraction absorbed from the starch formulation and the solution, the fractional absorption of FD4 180 min after administration of the CMC-Na formulation was increased by 2.06% and 2.52%, respectively.

### 3.2 The comparison of the nasal absorption of insulin from the solution and powder formulations in rats

**Fig 2:**
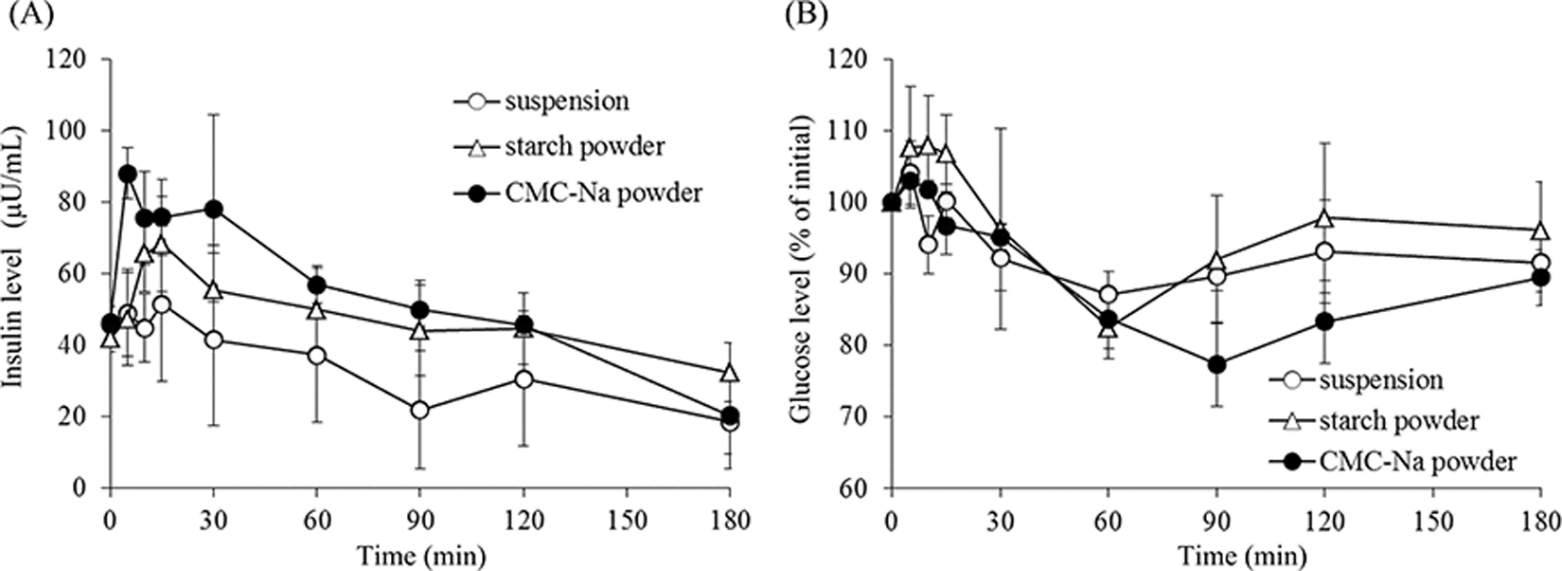
**The plasma level of (A) insulin and (B) glucose after nasal administration of various insulin formulations to rats.** Keys; ○, suspension; △, starch powder; ●, CMC-Na powder. Results are expressed as the mean ± S.E. of at least three experiments.

Fig 2 shows the profiles of the plasma concentration of insulin and glucose after administration of insulin as the suspension and powders to rats. Results regarding the concentration of insulin in the plasma are similar with those for FD4. As compared to the suspension, the level of FD4 in the plasma is increased after powder administration. A large decrease in the plasma glucose level was observed after nasal administration of the powder formulations, while the suspension of insulin showed a small hypoglycemic effect. By the addition of CMC-Na, the plasma concentration of insulin was increased, and plasma glucose was decreased significantly. Pharmacokinetic parameters (AUC, AAC, and *D*%) are summarized in Table 1. *D*% of insulin after application of the starch powder and the CMC-Na powder was increased by 6.1% and 11.3%, respectively, as compared to the suspension.

### 3.3 Cytotoxicity of CMC-Na in the nasal tissue

#### 3.3.1 Single administration

**Fig 3:**
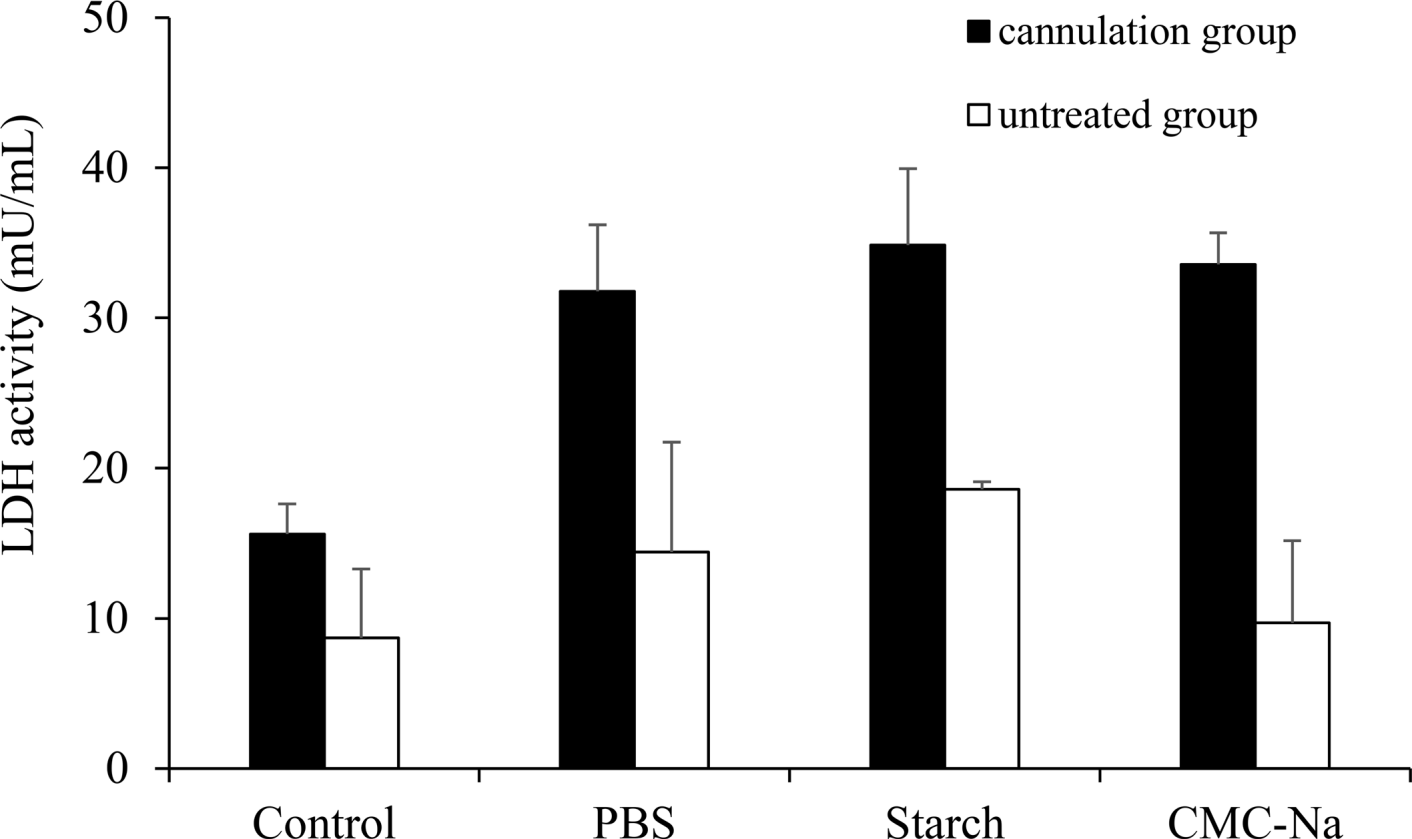
LDH activity in the nasal lavage fluid after single nasal administration of powder formulation containing CNC-Na. Keys: ■, cannulation group; □, untreated group. Results are expressed as the mean ± S.E. of at least three experiments.

Fig 3 shows the LDH activity in the nasal lavage fluid, which is an index of the damage to nasal epithelial cells^22, 23)^ after nasal administration of starch and CMC-Na to rats. The LDH activities of the cannulation group were 15.6 mU/mL (control group) and 31.8 mU/mL (PBS group), which were higher than those of the no-cannulation group. The LDH activities of the starch group and the CMC-Na group were not significantly different from that of the PBS group.

#### 3.3.2 Repeated administration

**Fig 4:**
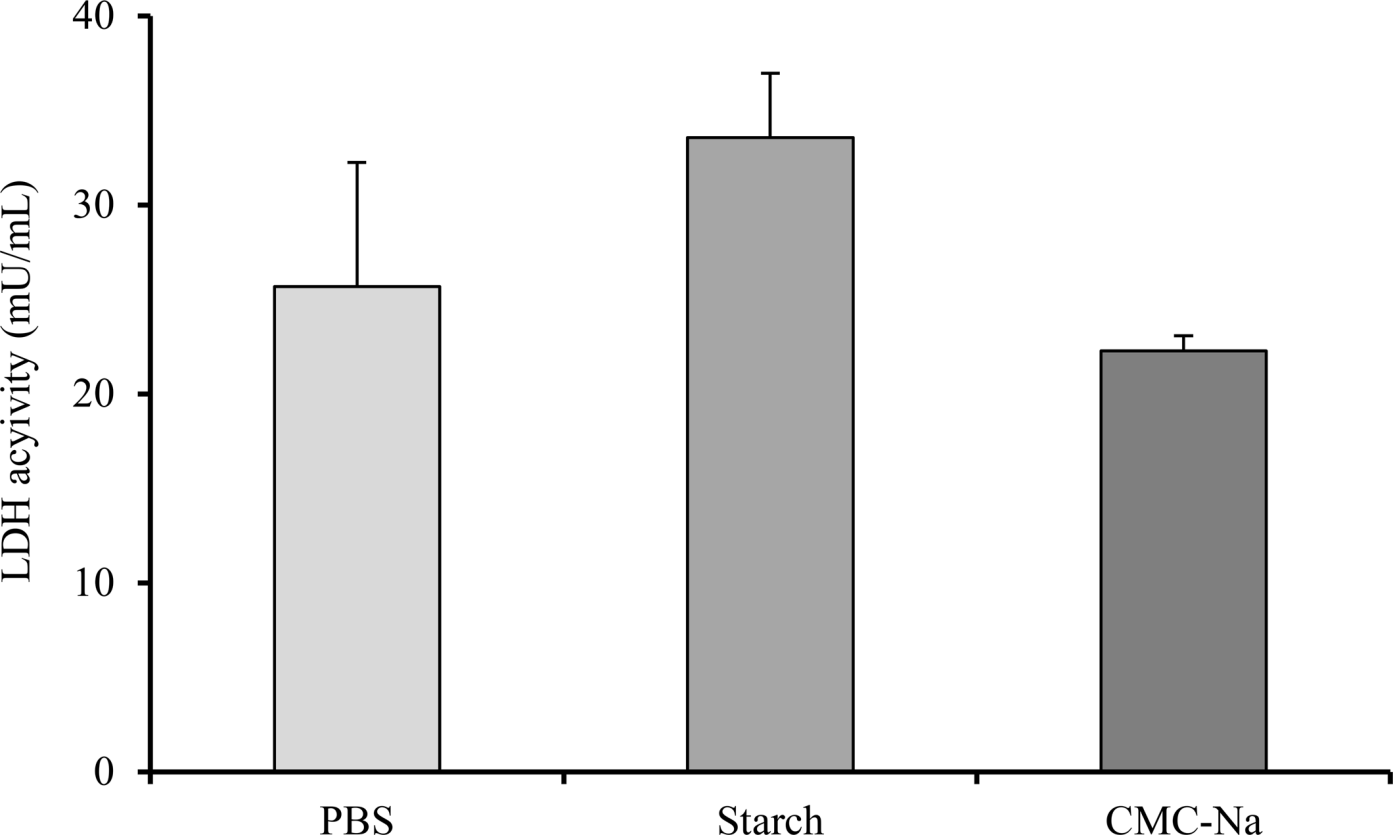
LDH activity in the nasal lavage fluid after repeated nasal administration of powder formulation containing CNC-Na. Results are expressed as the mean ± S.E. of at least three experiments.

Fig 4 shows LDH activity in the nasal lavage fluid after repeated administration. No significant change was observed in the LDH activity after repeated nasal administration.

### 3.4 Effect of CMC-Na on physiologic function of the nasal epithelium

**Fig 5:**
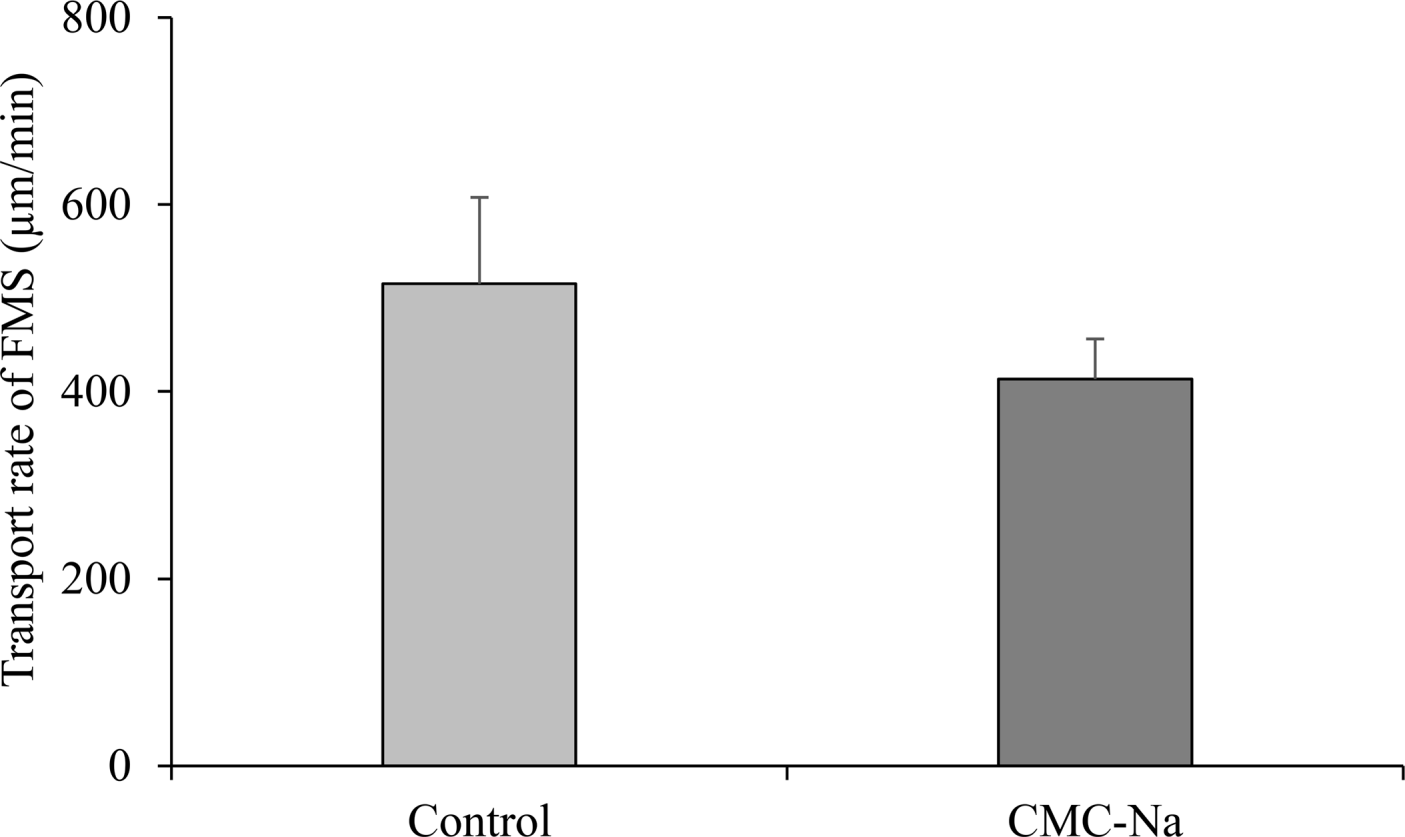
The effect of CMC-Na on the mucociliary clearance after nasal administration of the powder containing CMC-Na. Results are expressed as the mean ± S.E. of at least three experiments.

Fig 5 shows the effect of CMC-Na on mucociliary function after its intranasal administration. As shown in Fig 5, no significant difference was observed in the mucociliary clearance of the CMC-Na group.

## 4. Discussion

In general, the solid formulation has various advantages over the liquid formulation [13–16]. In the case of nasal drug delivery, the administration of a higher dose is one of the marked merits of the powder formulation. In addition, various excipients are usually used for the manufacturing of solid formulations. Excipients give functions to the formulation. In order to clarify the effect of excipients on the absorption of macromolecules, we focused on CMC-Na (sodium carmelose) in this study. Since CMC-Na has been used as a binder in manufacturing tablets and granules, its safety has been established.

Nasal absorption of the drug is generally quicker and higher than the gastrointestinal absorption. For example, propranolol, which is a highly permeable drug, shows a blood concentration profile following nasal application similar to that after intravenous administration [26]. In the case of nasal drug administration, a small volume of the drug solution (100 μL at most in humans) is applied. The drug solution can spread over the nasal epithelium without dilution. When the powder formulation is applied nasally, the powder can directly reach the surface of the nasal epithelium and dissolve in the small volume of fluid; thus, the drug concentration around the absorptive mucosa is very high. The drug concentration can be assumed to be saturated. On the contrary, after oral administration of solid formulations, the drug is dissolved in water with which the formulation is taken together (usually 200 mL). After dissolution in the stomach, the dissolved drug moves from the stomach along the intestinal tract, where it is diluted with gastric and/or intestinal fluid. The effective drug concentration around the mucosal epithelium after nasal administration of the powder is much higher than that after nasal application of the liquid and oral formulations, leading to the quicker and better nasal absorption from the powder formulation.

The surface of the respiratory mucosa is covered with mucus secreted from goblet cells. Some cells in the nasal epithelium are ciliated. Superficial mucus is translocated to the pharynx by ciliary beating for the clearance of noxious substances or infectious microorganisms trapped by the mucus layer out into the pharynx. Infectious microorganisms are killed by gastric acid. This respiratory function is called mucociliary clearance (MC), and is very important for the protection of the respiratory tract and for preventing infectious disease [27, 28]. The drugs applied into the nasal cavity are similarly cleared toward the pharynx by MC and swallowed into the stomach. Therefore, we believe that MC is one of the important factors determining the rate and extent of nasal drug absorption through the nasal residence of the drug [6, 7], although further studies are needed to elucidate the detailed mechanism underlying nasal drug absorption.

In the present study, FD4 was used as a model of non-degradable macromolecules. It was clarified that FD4 is hardly absorbed through the nasal mucosa from the liquid formulation. This is likely mainly because of the low permeability of FD4 through the nasal mucosa. The poor absorption of FD4 from the liquid formulation was enhanced by the administration of powder formulations. This change is likely based mainly on the higher concentration of FD4 around the nasal epithelium and partly on the decreased mucociliary clearance. Further enhancement of the absorption of FD4 is observed after nasal administration of the powder formulation to which CMC-Na is added. This finding is the result of the increase in the nasal residence of FD4 due to the increased viscosity of dissolved formulation by CMC-Na.

The results regarding the nasal absorption and the pharmacological potency of insulin were similar with those of FD4. This is likely due to the similar molecular weight (around 4 kDa) and the high solubility of these two macromolecules. The difference between FD4 and insulin is their stability. Generally, the enzymatic degradation of insulin in the nasal cavity decreases the nasal absorption and the potency of insulin. The degradation of insulin was unlikely the cause of the effect on the nasal absorption of insulin in our study. With regard to the enzymatic degradation of the peptide, the higher concentration of peptides/proteins around the nasal epithelium can saturate the activity of proteases to decrease the enzymatic barrier of the nasal epithelium. Therefore, we believe that this is the advantage of the powder formulation for the systemic delivery of peptide drugs, although additional studies are needed to examine the stability of peptide drugs in this formulation. The decrease in glucose levels after administration of insulin was greatly enhanced by the addition of CMC-Na to the powder formulation, which was in good agreement with the results of plasma insulin levels.

Understanding the local toxicity and side effects of CMC-Na is important for clinical application. The area where the powder formulation is applied is so small that CMC-Na may cause tissue damage. In this study, the damage to the nasal mucosal tissue was evaluated by the activity of released LDH after intranasal administration of CMC-Na as a powder. The LDH activity in the nasal lavage fluid was slightly but not significantly increased after nasal administration of CMC-Na, indicating that CMC-Na induces no damage in the nasal tissue, even after repeated administration. Since mucociliary clearance is an important physiologic function of the upper airway, attention should be paid to this function as an index of the undesirable effect of CMC-Na. In our previous manuscript, the easy and quantitative *in vitro* evaluation system for the ciliary function of the nasal epithelium using fluorescence microspheres was developed [20]. In the system used in this study, the translocation of the fluorescence microspheres on the excised rat nasal septum was recorded by a CCD camera. From serial photographs taken at 3 s intervals, the translocation speed of fluorescence microspheres (μm/min) was evaluated as an index of the nasal mucociliary function. This manuscript is the first to report the effect of the pharmaceutical excipient on nasal mucociliary function. According to the result from the study using this method, no significant change was observed in mucociliary clearance between the CMC-Na groups and the control group. CMC-Na does not affect the physiologic function of the nasal tissue, indicating the safety of CMC-Na as a nasal pharmaceutical excipient.

## 5. Conclusion

The present findings indicated that the powder formulation was effective in increasing the nasal absorption of FD4 and insulin. Furthermore, by addition of CMC-Na to the powder formulation, the absorption was further improved without any damage to the nasal tissue or any ciliary dysfunction. Nasal application of the powder formulation with the addition of CMC-Na may be a promising approach for improving the systemic nasal delivery of macromolecular drugs.
